# Tackling dengue fever: Current status and challenges

**DOI:** 10.1186/s12985-015-0444-8

**Published:** 2015-12-09

**Authors:** Taoufik Nedjadi, Sherif El-Kafrawy, Sayed S. Sohrab, Philippe Desprès, Ghazi Damanhouri, Esam Azhar

**Affiliations:** King Fahd Medical Research Center, King Abdulaziz University, Jeddah, Saudi Arabia; Special Infectious Agents Unit, King Fahd Medical Research Center, King Abdulaziz University, Jeddah, Saudi Arabia; UMR PIMIT (I2T team), University of Reunion island, INSERM U1187, CNRS 9192, IRD 249, Technology Platform CYROI, 2 rue Maxime Rivière Saint-Clotilde, La Reunion, 97491 France

**Keywords:** Dengue, *Aedes Aegypti*, Dengue hemorrhagic fever, Hemorrhagic shock syndrome, Genetic susceptibility, Vaccine development

## Abstract

According to recent statistics, 96 million apparent dengue infections were estimated worldwide in 2010. This figure is by far greater than the WHO prediction which indicates the rapid spread of this disease posing a growing threat to the economy and a major challenge to clinicians and health care services across the globe particularly in the affected areas.

This article aims at bringing to light the current epidemiological and clinical status of the dengue fever. The relationship between genetic mutations, single nucleotide polymorphism (SNP) and the pathophysiology of disease progression will be put into perspective. It will also highlight the recent advances in dengue vaccine development.

Thus far, a significant progress has been made in unraveling the risk factors and understanding the molecular pathogenesis associated with the disease. However, further insights in molecular features of the disease and the development of animal models will enormously help improving the therapeutic interventions and potentially contribute to finding new preventive measures for population at risk.

## Background

**Fig. 1:**
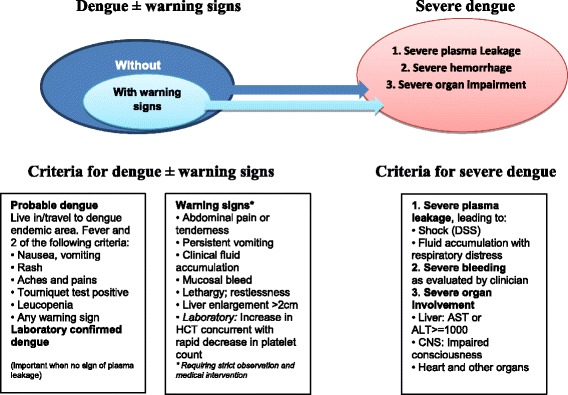
WHO dengue case classification (*Adopted from; Dengue Guidelines for diagnosis, treatment, prevention and control, New edn. Geneva: WHO; 2009*)

Dengue fever is a major cause of illness and death worldwide. The disease is caused by dengue virus which gets transmitted to humans by the bites of infected mosquitoes, *Aedes (Ae.) aegypti* and *Ae. albopictus* [1]. The disease represents a global health issue as it is endemic in around 100 countries, most of which are in tropical and sub-tropical areas. Over the last decades, the incidence rate and the geographic distribution of dengue have rapidly increased (almost 30-fold). Data from the World Health Organization (WHO) estimates up to 100 million cases of dengue fever each year [2]. However, a recent published work by Bhatt et al*.* (2013) suggested that the burden of dengue is far more than the WHO estimation and indicated that 390 million infections of dengue virus could have happened every year [3]. Changes in dengue epidemiology and the increase in incidence rates (with and without co-morbidities) have led the WHO to propose a new dengue classification system according to disease severity (Fig. 1) [2].

### Etiology and mode of transmission

Dengue fever is caused by infection with dengue virus (DENV). The DENV is a vector-borne virus transmitted to humans primarily by bites from two mosquito species, *Ae. aegypti* or *Ae. albopictus*. DENV is a single positive-stranded RNA virus belonging to Flavivirus genus of the *Flaviviridae* family and has 4 major serotypes (DENV 1–4) that are antigenically distinct from each other. Each DENV serotype is phylogenetically distinct suggesting that each serotype could be considered a separate virus [4]. Three dengue serotypes out of four (DENV 1–3) have been found in Middle Eastern countries including Saudi Arabia and Yemen. Interestingly, DENV-1 strain isolated in Saudi Arabia exhibited a high genetic similarity with DENV-1 strain isolated from Asian population, suggesting a widespread of the Asian genotype, probably through Asian pilgrims [5, 6]. A recently published article has unveiled a new serotype (DENV-5), to be added to the existing ones [7]. This discovery is still controversial and little-known enough to conclude how the 5^th^ dengue serotype might add to the burden associated with dengue infection.

Mosquitoes transmit the virus by feeding on blood of infected persons. At first, the virus infects and replicates in the mid-gut epithelium of the mosquito and then spreads to other organs until it reaches the salivary glands after 10–14 days where it can be inoculated to another person during subsequent blood meal. Vertical transmission of DENV in mosquitoes, i.e. from mosquito to larvae has been reported by a number of research groups. In India, Angel & Joshi (2008) reported the detection of dengue virus by indirect fluorescence antibody test (IFAT) in laboratory reared mosquitoes originating from larvae collected from urban and rural areas [8]. A similar study was conducted in Brazil by Martins et al. (2012) and confirmed the isolation of DENV-type 3 in *Ae. albopictus* larvae and DENV-type 2 in *Ae. aegypti* larvae [9]. Similar findings were also reported in Mexico [10] and Indonesia [11]. On the other hand, mother-to-infant transmission of dengue virus via cord blood or breast milk remains controversial [12–14].

### Clinical manifestations

Based on the results from several studies, the WHO has launched a new dengue classification. This classification divides dengue cases into a) cases with/without warning signs and b) severe dengue cases [2]. However, it is important to note that numerous research groups have debated the rational of this classification as it does not fit their unique local settings. The criteria for dengue case classification are presented in Fig. 1.

Clinically, dengue infection has a broad spectrum of features. The vast majority of cases are asymptomatic and passes unnoticed. Typically, the symptoms start to be prominent after an incubation period of 3–10 days [15]. The severity of the clinical manifestations varies from mild symptoms to severe life threatening symptoms in the case of dengue hemorrhagic fever (DHF) and dengue shock syndrome (DSS) [16]. Predicting the progression of the mild signs to a severe DHF/DSS remains a challenge due to non-specificity of clinical presentation and the incomplete understanding of pathophysiology of the disease and its underlying molecular mechanisms.

### Dengue with warning signs

The early signs of the disease are non-specific. According to the WHO classification (2009), DF is characterized by febrile episode (≥40 °C for 2–7 days) frequently associated with rash, nausea, vomiting, and headache. Although the disease affects people of all ages from infancy through to adulthood [17], epidemiological data showed that children tend to tolerate this phase of illness better than adults [18]. The persistence of the aforementioned symptoms and appearance of other symptoms, such as abdominal pain, mucosal bleed, and lethargy and restlessness can be seen 3–7 days later. Laboratory analysis of mild dengue fever cases usually shows abnormal leukocyte counts and moderate elevation of the hepatic amino-transferase enzyme activity [19]. The emergence of these symptoms is a warning sign for disease progression to severe form (DHF/DSS) if therapeutic intervention is not undertaken. At this stage clinical intervention and continuous surveillance are imperative to prevent vascular leakage, especially in an endemic area.

### Severe dengue

This form of dengue infection can be attributed to any of the four known serotypes DENV 1–4. The likelihood of developing DHF/DSS is high in patients who have experienced dengue infection in the past with heterogeneous serotype [20]. About 5–10 % of patients progress to develop a severe DHF/DSS which can be fatal unless treated promptly [21]. This form develops at a late stage of DF, where patients may go through defervescence phase characterized by a sudden drop of body’s temperature. This phase is also distinguished by severe bleeding, particularly bleeding from the gastrointestinal tract (black, tarry stool), and thrombocytopenia (<50,000/mm3), which may affect up to 50 % of DHF cases [22]. Interestingly, there was an observed negative correlation between the severity of DHF and the level of platelets in the blood. The exact mechanism of this correlation has yet to be delineated. The drop of platelet counts and the loss of their functionality lead to a vascular fragility increasing the risk of hemorrhage and plasma leakage [23]. It has been suggested that during acute phase of the infection DENV replicates quickly in platelets, as this is very critical for virus survival and dissemination [24, 25].

The existence of other symptoms such as retro-orbital pain, maculopapular rash, petechiae, or bleeding from the nose or gums will help making definitive diagnosis for DF [25]. Evidence of plasma leakage in various body cavities such as the pleural cavity and the peritoneal cavity, associated with profuse perspiration, adynamia, and sometimes fainting are signs of rapid progression to shock. Subsidence in systolic pressure and hypotension may result in profound shock, known as dengue shock syndrome (DSS). The duration of DSS for a long time might predispose to further complications such as massive bleeding, disseminated intravascular coagulopathy (DIC), respiratory failure, multi-organ failure, and infrequently encephalopathy leading to death [26, 27]. It has been proposed that case fatality related to DHF may reach 15 % of all cases, however, proper medical care and symptomatic management can reduce mortality rate to less than 1 % [28].

### Diagnosis

An early and accurate laboratory diagnosis of dengue infection is of paramount importance in the management of the disease. It has been estimated that the number of misdiagnosed dengue cases could reach a record ratio of 50 % of all cases, mainly due to a large disparity of dengue signs and symptoms which overlap with the symptoms of other viral infections, especially for persons living in or traveling to endemic areas of tropical infectious diseases. Dengue fever should be distinguished from other illnesses which share similar symptoms such as chikungunya, Mayaro fever, Ross River fever, West Nile fever, Zika fever, yellow fever and viral hemorrhagic fevers [4]. Until the antiviral vaccine becomes available, the prevention of severe cases and cut-down of the economic burden of the disease rely enormously on early and accurate diagnosis. The latter is made possible through the availability of several diagnostic laboratory and virological tests.

The onset of later stage symptoms of the illness can be overwhelming and more pathognomonic. Nonetheless, based on WHO classification schemes, the appearance of leukopenia in patients with febrile illness is a major consideration in making diagnosis of dengue infection [29]. Overall, there is an urgent need to reduce dengue morbidity and mortality by improving the diagnosis and molecular analysis of emerging dengue virus. Thus far, two diagnostic modalities have been applied to detect the disease at an early stage. The first one is a direct method targeting the acute phase of dengue disease, which is based upon detection of genomic RNA by RT-qPCR or soluble NS1 by antigen capture in blood samples from viremic patients. The second is the indirect method that relies on serological tests to detect dengue-related immunoglobulins par Mac-ELISA for the capture of specific IgM or indirect ELISA for the capture of anti-DEN IgGs [30–34].

### Genetic alteration/susceptibility to dengue infection

Several risk factors have been associated with dengue infection and its progression to severe DHF/DSS forms. Recent advances in molecular biology have revealed that the genetic makeup of the three elements of dengue infection (the virus, the vector, and the host) plays a primordial role in the pathogenesis of the disease and could potentially contribute to the DHF progression [19, 24, 35]. Hence, an in-depth analysis of genetic variability including polymorphism and mutations could be beneficial in identifying the possible factors and mechanisms of disease development [36]. The list of host’s genetic factors that confer susceptibility or resistance to dengue infection is summarized in Table 1.

**Table 1:** Genetic susceptibility

Gene	Function	Reference
FcγRIIa	Protected from DHF	*Am. J. Trop. Med. Hyg.* 2002*,* **67**(1):
Vitamin D receptor (VDR)	Associated with resistance to severe dengue	Human Immunology. 2012, **73**: 1194–1199.
IL-10	Associate with dengue disease severity	Plos one. 2012, **7** (11), e50387.
TNFa	Associate with dengue disease severity	Plos one. 2012, **7** (11), e50387.
TGFb	Associate with dengue disease severity	Plos one. 2012, **7** (11), e50387.
JAK1	Strongly association with DHF	Eur. J. Human Genet. 2010, **18**: 1221–1227.
HLA class I alleles A*01, A*0207, A*24, B*07, B*46, B*51	DHF development	Clin Microbio Rev. 2009, **22** (4): 564–581., Tissue Antigens **60**:309–318.
HLA class II alleles DQ*1, DR*1, DR*4	DHF development	Clin Microbio Rev. 2009, **22** (4): 564–581.
Glucose-6-phosphate dehydrogenase	Increase DENV replication	Blood Cells Mol. Dis. 2009. **42**:267–278.
DC-SIGN	Associated with DENV infection	Med Sci (Paris). 2005, **21**: 905–906.
TAP	Prevent DHV/DSS	Scand J Immunol **67**:618–625
MBL	Protect against DENV	Hum Immunol **69**: 122–128.
CTLA-4	Increases the risk of DHF.	Clin Immunol **131**: 404–409.
MICB	Associated with severe forms of dengue	Nat Genet. **43**(11): 1139–1141
PLCE1	Associated with severe forms of dengue	Nat Genet. **43**(11): 1139–1141
ABO blood group	Increases the risk of DHF	J Inf Dis **195**:1014–1017
HPA2	Enhance susceptibility to DHF	Hum Immunol **68**:973–979
IFN-γ	Enhance severity of dengue disease	FEMS Immunol Med Mic 2000; **28**: 183–188.
IL-6	Associated with DSS	PLoS Negl Trop Dis. 2013, **7**(9): e2412.
VCAM-1	Associated with severe dengue	Trop Med Health. 2014, **42**(4): 137–44.
Oligoadenylate synthetases (OAS)	Enhance susceptibility to dengue infection	Infection, Genetics and Evolution 2013, **14**: 390–395.

List of genes that confer susceptibility to dengue infection. The journal volume is given in bold

### The mosquito

Like most arboviruses, DENV infect different organs of the mosquito, including the salivary glands and the central nervous system. Mosquito infection elicit behavioral changes including increase of the probing time which lead to host interruption that might lead to wider spread of the virus [37]. It has been demonstrated that DENV infection induced the expression of cathepsin-B, a putative cystatin, and a hypothetical ankyrin repeat-containing protein genes [38]. The latter could alter the efficiency of virus replication in the salivary gland. This study has shown that modulation of *OBP10* and *OBP22* genes expression as well as DENV infection-responsive odorant-binding protein genes increase the time length for initiation of probing before a successful blood meal, resulting in changes in the host seeking behavior of the mosquito. Comparative analysis of the salivary gland transcriptomes of native and DENV-infected *Ae. aegypti* identified a number of differentially expressed genes related to sugar/protein digestion enzymes, immunity related genes and blood meal acquisition enzymes that might have an impact on the efficiency of viral replication or mosquito feeding behavior. This study showed that DENV infection alter the expression of key host-seeking genes in the mosquito’s main olfactory organs and the antennae [38].

Recent updates have indicated that resistance of *Ae. aegypti* to conventional insecticides is related to different mechanisms, one of which is associated with genetic abnormalities within the vector’s genome. Single point mutation in the voltage-gated sodium channel gene at position 1534 (*F1534C*) resulting in phenylalanine to cysteine substitution in *Ae. aegypti* confers resistance to permethrin. This mutation is widespread in this vector in Southeast Asia and Latin America [39, 40]. It has also been reported that a single amino acid substitution Valine to Glycine at position 1016 in domain II, segment 6 of the voltage-gated sodium channel gene was associated with less sensibility of *Ae. aegypti* to deltamethrin in Thailand [41].

### Human susceptibility to dengue disease

Numerous multi-disciplinary studies confirmed that race, young age, virus strain, female sex and high body-mass index correlate well with increased burden of dengue infection. The observation that people of African background are less likely to develop DHF/DSS compared to their Caucasian counterpart has led to the suggestion that host genetic variability has a major impact on the clinical manifestations of dengue infection [42, 43]. Thus, a closer consideration of human genes regulating the severity of dengue infection, especially genes associated with the immune response, might help in controlling disease spread and improve the acute symptoms of the infection. A number of studies have investigated the relationship between the host genetic polymorphisms and DENV infection (Table 1).

A single nucleotide polymorphism (SNP) in the promoter of CD209/DC-SIGN was associated to increased risk of developing dengue fever [44]. Association studies have successfully identified a link between polymorphisms in the *human major-histocompatibility-complex (HLA) class I/II genes* and *non-HLA* host genetic factors and severity of dengue disease [45–47]. Polymorphisms of the *TAP1* and *TAP2* genes could be directly associated with the risk of developing dengue disease among the primary-infected individuals [48]. Both *TAP1* and *TAP2* are located within the *MHC class II* region and homozygosity of the *TAP1* at position 1333 and 1637 and for *TAP2* at position 2379, respectively, was found to protect against developing severe forms of dengue [46].

In an independent study [49], the authors showed that single nucleotide polymorphism of the oligoadenylate synthetase genes (*OAS1, 2 and 3*), of the OAS/RNase L antiviral immune system, enhance susceptibility to clinical outcomes of dengue infection. An association between the severity of the disease and other genes including human leukocyte antigen class I and class II genes, tumor necrosis factor-alpha, *FcGRIIA*, vitamin D receptor, transporters associated with antigen presentation, and *JAK1* has also been proposed [50]. The importance of *Vit-D* in DENV pathogenesis was concluded from newly-gathered data showing that *Vit-D* impairs DENV replication and polymorphism of *Vit-D* gene increases the expression of both CD209/DC-SIGN and FcGRIIA receptors that enhance DENV entry in the target cells [51, 52].

In another study [53], the authors have successfully applied genome-wide association study (GWAS) approach to identify loci that confer susceptibility to severe forms of dengue disease. The investigators used samples from 2008 children affected with severe dengue infection against 2018 population control cases in Vietnam. The data showed that SNPs at two loci, *MICB* and *PLCE1*, significantly increased the likelihood of developing DSS in children. This finding was further validated in an independent cohort of 1737 cases and 2934 controls [53]. A SNP in the *MICB* gene coding for the MHC class I polypeptide-related sequence B, an inducible activating ligand for the NKG2D type II receptor of immune cells could alter the protective role of natural killer and CD8^+^ T cells in the host responsiveness to DENV at the early stage of infection [54, 55]. On the other hand, *PLCE1* plays a primordial role in maintaining intact vascular endothelial cell barrier function, hence, polymorphism of the *PLCE1* gene may lead to blood vessels leakage and circulatory hypovolemia during DSS [56].

Other host candidate genes have also been associated with early onset dengue disease. Among these genes, there were receptors/attachment factors for DENV linked to immune system and inflammatory response. The chemokines CXCL10, CXCL11 and its respective chemokine receptor CXCR3 were reported as biomarkers for severe form of dengue infection [57]. These results are in agreement with recent emerging data indicating strong association between CXCL10, CXCL11 and CXCR3 and vascular permeability [58]. The three genes are components of the NF-kB pathway and are involved in the pathogenesis of SARS and West Nile virus encephalitis [59, 60]. Cerney et al*.* (2014) interrogated the effect of DENV on the first point of human contact which is skin cells. The authors demonstrated an increase expression of IFN-β, STAT-1 and CCL5 in a susceptible population of skin dendritic cells (DC) which may facilitate the spread of DENV in the blood [61]. This process depends enormously on vector-derived salivary factors inoculated on the skin cells [62].

### Current status of dengue vaccine development

Till-date, there is no effective, commercially available, therapy/vaccine for dengue virus. Numerous groups have already made intensive efforts and made good progress to develop a safe, affordable and effective vaccine against all serotypes for global public health [63–69]. Vaccines which are being developed use various approaches such as live attenuated viruses, inactivated viruses, subunit vaccines, DNA vaccines, and chimeric viruses using yellow fever vaccine and attenuated dengue viruses as backbones (Table 2).

**Table 2:** Current status of dengue vaccine development

No	Strategy	Developer (s)	Current status
1	Live attenuated yellow fever 17D/DENV chimeric vaccine	Sanofi-Pasteur	Phase 3 trials with a tetravalent formulation in DENV endemic countries
2	PDK cell-passaged, live attenuated vaccine	WRAIR/GSK	Phase 2 trials with a tetravalent formulation in endemic countries
3	Live attenuated DENV Delta-30 mutation and intertypic DENV chimeric vaccines	NIH/Johns Hopkins	Phase 1/2 trials with monovalent formulations completed; tetravalent
			phase 1 initiated
4	Dengue prM-E DNA vaccine	NMRC	Phase 1 with monovalent vaccine completed
5	Recombinant 80 % E subunit antigen vaccine	Hawaii Biotech/Merck	Phase 1 with monovalent vaccine initiated
6	Purified inactivated vaccine	WRAIR	Phase 1 with monovalent vaccine initiated
7	Live attenuated chimeric DENV vaccine	CDC	Phase 1 with monovalent vaccine initiated

Abbreviations: PDK, primary dog kidney cells; WRAIR, Walter Reed Army Institute of Research; GSK, GlaxoSmithKline Biologicals; NIH, National Institutes of Health; prM-E, premembrane-envelope; NMRC, Naval Medical Research Center; CDC, Centers of Disease Control and Prevention

### Live attenuated yellow fever 17D/DENV chimeric vaccine

Currently, only one tetravalent vaccine against dengue virus, developed by Sanofi-Pasteur (France) has reached phase III clinical trial and is expected to be launched in 2015. This vaccine is based on the production of four chimeric live dengue-yellow fever viruses in which the yellow fever (YF) 17D vaccine sequences encoding the envelope proteins prM and E genes were substituted by the prM and E genes from DV of serotype 1, 2, 3, or 4 in a molecular clone of YF-17D [69]. This vaccine was produced and tested over 6000 people using four dengue virus isolates from Indonesia and Thailand. This candidate vaccine was found to be attenuated and stable in animal models with respect to plaque size and yellow fever virus neurotropism [70]. Results of the clinical trials showed no adverse effects except moderate injection site pain, headache, and myalgia. Another randomized, controlled trial was launched using a total of 4002 Thai school children to investigate the efficacy of a recombinant, tetravalent vaccine for dengue virus and only 134 dengue cases were reported [71]. Phase I trial of the vaccine in the Philippines showed that the seropositivity increased gradually (53, 72 & 92 %) after 1–3 vaccinations against all four serotypes as compared to control group. The most promising results were observed in children 2–5 years old who exhibited high levels of reactivity of 91, 100, 96, 100 % for DENV 1–4; respectively [72].

Another placebo-controlled trial was conducted on 10,275 children from Vietnam (vaccine, *n* = 6851 Vs placebo, *n* = 3424) to determine the clinical efficacy and safety of CYD-TDV. The results demonstrated virologically-confirmed cases in 47 % of the vaccine group as compared to the control group (53 %). The efficacy was achieved in up to 56.5 % (95 % CI 43.8–66.4). These findings indicated that the vaccine is highly efficacious with good safety profile when three injections were given to children with age group 2–14 years at 0, 6 and 12 months intervals [73]. The data emerging from another randomized phase II trial in India indicated that the vaccine has no serious adverse events and the immunogenicity and safety of CYD-TDV were satisfactory [74]. A pilot study carried out in five Latin American countries where more than 20,000 children aged 9–16 were recruited to receive either the CYD-TDV vaccine or placebo. The results on efficacy (60.8 %) and safety profiles were consistent with the previous findings [74, 75]. Interestingly, the vaccine efficacy (80.3 %) against hospitalization for dengue was promising and represented a step forward to developing an effective dengue vaccine [75].

### Live attenuated DENV delta-30 mutation and intertypic DENV chimeric vaccines

Other candidate dengue vaccines have been developed in USA by the Johns Hopkins University and National Institute of Allergy and Infectious Diseases (NIAID) and have reached advanced clinical trials [65]. Four live-attenuated DENV/delta-30 were generated each containing 30 nucleotides deletion of the 3’-untranslated region of genomic RNA (delta-30). These vaccines efficiently impaired viral growth in human liver carcinoma cells [76]. To improve the attenuation of DENV-2/delta-30 and DENV-3/delta-30, chimeric DENV were developed by substitution of the *prM-E* gene region of DENV-4/delta-30 virus with the prM-E genes of DENV-2 and DENV-3 [72, 77]. The results from phase I clinical trial showed that all four live-attenuated DENV/delta-30 are safe and immunogenic with minor side effects such as faint rash and transient leucopenia only after higher dose [78, 79].

### Dengue-measles vaccine

Dengue virus serotype-1 antigen was expressed in a vector based on pediatric live-attenuated Schwarz measles vaccine (MV) by using the envelope domain III (EDIII) fused with the ectodomain of the membrane protein (ectoM). After immunization, long-term production of DENV-1 serotype-specific neutralizing antibodies was observed in measles virus susceptible mice [80]. A new strategy was evaluated based on single minimal tetravalent DENV antigen expression using viral vector derived from pediatric live-attenuated measles vaccine (MV). A recombinant MV vaccine construct was developed using envelope domain III (EDIII) and ectodomain of the membrane protein. The neutralizing antibodies were induced against all four serotypes of dengue virus after two injections in mice susceptible to MV infection. A strong memory neutralizing response was observed against all four serotypes in immunized mice after inoculation with live DENV from each serotype [81].

### Dengue prM-E DNA vaccine

A naked DNA-based candidate vaccine against DENV has been developed by the Naval Medical Research Center [67, 82, 83]. The genes encoding prM and E of DENV were cloned into a shuttle vector under the transcriptional control of human cytomegalovirus (CMV) promoter. The results of phase I clinical trial showed no adverse effects except mild injection site pain, swelling, and fatigue. After second dose, strong IgM and IgG antibody response was observed which favors the safety profile of this vaccine. To get a better immunogenicity profile, a vaccine based on lipid adjuvant Vaxfectin (Vical Incorporated, San Diego, USA), was developed and the results demonstrated good protection profile against DENV compared to DNA alone [84]. Based on this technology, different groups have developed other candidate vaccines and achieved good protection in mouse models using envelope glycoproteins prM and E, the non-structural protein NS1 and the helicase/protease NS3 as vaccine antigens [85–87].

### Purified inactivated vaccine (PIV)

The first purified inactivated vaccine was developed with aluminum hydroxide (alum) adjuvant and tested in mice and rhesus macaques in the mid-1990s, by Walter Reed Army Institute of Research against dengue 2 serotype and good virus protection was reported after two doses [88, 89]. Using similar technology, second generation Japanese encephalitis (JE) PIV vaccine was developed [90, 91]. Currently, a new JE vaccine (Ixiaro; Novartis Vaccines) has been approved for use in many countries, including the USA [92]. Another dengue vaccine (dengue 1 PIV), recombinant subunit dengue E glycoprotein antigen (r80E) was also developed and has entered phase I clinical trial [93–95]. The Centers for Disease Control and Prevention (USA) have also developed a live-attenuated vaccine named DENVax, which was found to be highly immunogenic in both children and adults and has currently entered phase I clinical trial in the United States [96, 97]. Recently, a novel third generation approach is being used to develop a vaccine containing recombinant subunit E domain III (*ED3*) and the results of laboratory tests have shown the development of potent neutralizing antibodies in a mouse model [98–100]. Using the same technology, a tetravalent vaccine was developed and expressed in *Pichia pastoris* by splicing and using flexible pentaglycyl linkers of the four EDIII. The observed results showed that this antigen elicit specific antibodies against all four DENV serotypes in BALB/c mice [101].

### Lessons from animal models

Animal models are very useful for vaccine test development. The lack of animal models significantly hampered the development and efficacy testing of dengue vaccine. Currently only rhesus macaques and *Aotus* monkeys are being used for testing the vaccine before clinical trials are initiated [62]. The D1ME100 vaccine was evaluated in both *Aotus* monkeys and rhesus monkeys, and found to be immunogenic with 80–95 % protection against dengue infection [102, 103]. Porter et al. (2012) demonstrated that injection of non-human primate with three doses on day 1, 28 and 84, with tetravalent dengue DNA vaccine Vaxfectin-adjuvanted, was more efficient against live dengue-2 virus compared to control animals. This finding support initiation of Vaxfectin-adjuvanted phase I clinical trial [84].

Successful induction of immune response was obtained in mice and rhesus monkeys to the vaccines developed using dengue 4 prM-E, dengue 1 *prM-E-nonstructural (NS)1*, and dengue 2 NS3 antigens, and PIV adjuvanted with alum [85, 86]. Centers for Disease Control and Prevention (Fort Collins, CO), Hawaii biotech, and Simmons developed different vaccines that showed good immunogenicity in animal models [104]. Similarly, the psoralen/UV inactivation dengue vaccine was found to be more immunogenic and protective against dengue serotype 1 virus in *Aotus* monkeys [105].

### Antiviral therapy

Thus far, there are no antiviral drugs available to treat dengue fever; therefore the community will continue to depend on the control of the mosquito vector as the main route to prevent the spread of disease. Alternative approaches have been utilized against flaviviruses by targeting and inhibiting virus entry and the essential elements used in virus replication, nonstructural proteins, RNA polymerase, and proteases. The most important target elements include NS3 helicase nucleoside triphosphatase (NTPase/RNA 5’ triphosphatase (RTPase), NS5 methyl transferase/RNA-dependent RNA polymerase, and NS3/NS2B protease [106–108].

RNA interference (RNAi) technology is also being used to impair virus replication against respiratory syncytial virus, hepatitis viruses, influenza virus, poliovirus and HIV [109, 110]. Low molecular weight phenolic compounds such as flavonoids and phytochemicals isolated from plants were previously tested and are being used for anti-dengue therapy [111, 112]. An anti-viral inhibitory effect ranging from 50–75 % against DENV replication was observed when methanolic extracts of *Momordica charantia* and *Andrographis paniculata* were used in cultured primate cells [113].

Several attempts have been made in the past to tackle dengue through elimination of *Ae. Aegypti.* The most successful experiences were related to vector control programs adopted in Cuba and Singapore. The programs were based on intensive insecticidal treatment and reduction of the availability of Aedes larval habitats [18, 114]. Unfortunately, lack of sustainability of these stringent measures led to reappearance of dengue outbreaks.

Recently, a novel form of biological control of dengue transmission has been developed and is currently being applied. This is based on the development of genetically modified (GM) mosquitoes infected with a bacterium known as Wolbachia to combat dengue infection. This bacterium blocks replication of the virus inside the mosquito and prevents its transmission to humans [115]. In 2012, 10 million GM male mosquitoes were released in the wild to decrease the number of *Aedes* mosquitoes and reduce the rate of dengue transmission. A closer monitoring of the insects revealed that over 85 % of the eggs were Wolbachia-positive which indicated that GM-mosquitoes were overriding wild-mosquitoes resulting in decreased virus transmission [116]. In an initiative to eradicate dengue fever, scientists from Australia, are leading Eliminate Dengue (ED) program which involves community engagement as a key component in this program. Since the program kicked off in 2011, millions of Wolbachia mosquitoes were released across the North Queensland city—Australia. Based on the promising results obtained from local trial, Eliminate Dengue became an international research program across countries affected by dengue including Australia, Vietnam, Indonesia, Brazil and Colombia [117, 118].

### Targets of antiviral therapy

Dengue infection can be prevented by alternative approaches. The first one includes blocking virus entry into cells which is mediated by the viral envelope glycoprotein E via receptor-mediated endocytosis [119]. Dendritic cells, monocytes, and macrophages are the main targets of DENV infectious entry. The second approach involves blocking virus attachment to specific cellular receptors expressed on immune cells, liver cells, and endothelial cells.

### Fusion and glycosidase inhibitors

Small molecules and peptides targeting the hydrophobic pocket of the envelope E glycoprotein are characterized as inhibitors of virus entry. Nicholson et al. (2011) explored the inhibitory effects of DN59 and 1OAN1, peptide entry inhibitors. The authors demonstrated that DN59 and 1OAN1 can effectively block antibody dependent enhancement (ADE) *in-vitro* suggesting that entry inhibitors are potential candidates to prevent development of DHF/DSS [120]. Two other compounds have also been shown to qualify as potent inhibitors of dengue virus infection are imino-sugars deoxynojirimycin and castanospermine [121]. These compounds are natural alkaloids derived from the black bean and act as inhibitors against all 4 dengue serotypes by disrupting the folding pathways of the envelope glycoproteins prM and E [122].

### Carbohydrate-binding agents

Various types of carbohydrate-binding agents, isolated from different organisms, have been shown to have anti-viral activities. Three plant lectins, *Hippeastrum hybrid* agglutinin, *Galanthus nivalis* agglutinin and *Urtica dioica* agglutinin isolated from amaryllis, snowdrop and stinging nettle respectively were found to be potent inhibitors of DENV-2 infection by inhibiting viral replication [123].

### Heparan mimetics

Heparan sulfate (HS) is a putative receptor for DENV which interacts with domain III of the E-protein. Virus entry can be blocked by targeting the E-protein-HS interaction with soluble GAGs and other highly charged HS [124]. Fucoidan was isolated from marine algae and showed antiviral activity against DENV-2 in BHK cells [125]. Similarly, carrageenan and DL galactan, sulfated polysaccharides from red seaweeds, exhibited strong antiviral activity against DENV-2 and DENV-3 but a very weak activity against DENV-4 and DENV-1. Furthermore, two *α*-D-glucans were isolated from a Chinese herb and demonstrated high anti-DENV-2 activities in BHK cells [112, 126].

## Conclusions

Dengue fever represents a real economic burden especially in affected countries. Extensive efforts are needed to tackle disease spread and reduce the mortality rates and the associated healthcare cost. There is a need for more scientific research which we believe is a key route to provide further insight in the pathogenesis of dengue infection and help understanding the underlying molecular mechanisms associated with progression to the severe forms of the disease (DHF/DSS). This will be a step forward to develop an adequate preventive vaccine and effective treatment.

## Abbreviations

DENV: Dengue virus
DF: Dengue fever
DHF: Dengue hemorrhagic fever
DSS: Dengue shock syndrome
DC: Dendritic cells
